# Housing Shortages in Urban Regions: Aggressive Interactions at Tree Hollows in Forest Remnants

**DOI:** 10.1371/journal.pone.0059332

**Published:** 2013-03-21

**Authors:** Adrian Davis, Richard E. Major, Charlotte E. Taylor

**Affiliations:** 1 School of Biological Sciences, University of Sydney, Sydney, NSW, Australia; 2 Australian Museum, Sydney, NSW, Australia; Stanford University, United States of America

## Abstract

Urbanisation typically results in a reduction of hollow-bearing trees and an increase in the density of particularly species, potentially resulting in an increased level of competition as cavity-nesting species compete for a limited resource. To improve understanding of hollow usage between urban cavity-nesting species in Australia, particularly parrots, we investigated how the hollow-using assemblage, visitation rate, diversity and number of interactions varied between hollows within urban remnant forest and continuous forest. Motion-activated video cameras were installed, via roped access to the canopy, and hollow usage was monitored at 61 hollows over a two-year period. Tree hollows within urban remnants had a significantly different assemblage of visitors to those in continuous forest as well as a higher rate of visitation than hollows within continuous forest, with the rainbow lorikeet making significantly more visitations than any other taxa. Hollows within urban remnants were characterised by significantly higher usage rates and significantly more aggressive interactions than hollows within continuous forest, with parrots responsible for almost all interactions. Within urban remnants, high rates of hollow visitation and both interspecific and intraspecific interactions observed at tree hollows suggest the number of available optimal hollows may be limiting. Understanding the usage of urban remnant hollows by wildlife, as well as the role of parrots as a potential flagship for the conservation of tree-hollows, is vital to prevent a decrease in the diversity of urban fauna, particularly as other less competitive species risk being outcompeted by abundant native species.

## Introduction

Urbanisation typically results in heavy fragmentation of the landscape, creating a complex matrix of remnant vegetation, housing and industrial estates surrounded by continuous native forest [1], [2]. This re-structuring of the landscape can result in changes to the composition and richness of biotic communities and changes in species’ distributions [3], [4]. Birds, in particular, have been a major focus of urban ecological research ([5]) and it is well documented that some urban bird populations have greater densities than populations in their original habitat [6]–[8]. In Australia, avian communities within some urban regions now comprised both a higher abundance and a more diverse assemblage of some parrot species compared to that which was historically present [3], [7]. Few studies, however, have focused on the impact of urbanisation on birds with specific nesting requirements, such as cavity-nesting species.

As parrots are cavity-nesters, tree hollows may become a critical resource that may strongly influence the ability of some species to sustain urban populations. Not all cavities are suitable for some species to utilise [9] and when these cavity types are limited, intense interspecific competition may occur as different species compete for the same type of resource [10]–[13]. High levels of aggression have been observed at tree hollows amongst conspecifics and interspecifics[12], [14]–[16]. Guarding of tree hollows and other aggressive interactions, including the killing of interspecific chicks, has been observed both at the hollow and within buffer zones established around the hollow-bearing tree [12], [15], [16].

Remnant vegetation within urban landscapes has been shown to contain half the number of hollow bearing trees per 2 hectares than continuous forest (Davis, unpubl. data). The continued loss of hollow-bearing trees due to land clearing, senescence, and suppression of abiotic processes (such as wild fire) that promote natural hollow development, may lead to a potential shortage of hollows in urban landscapes, particularly in areas where hollow development is slow [17], [18]. Unlike in Europe, and North and South America, where primary hollow development frequently occurs through active excavation by woodpeckers, hollow development in Australia is a secondary process and dependent on insect damage and/or fungal decay following damage to the tree [19]–[24]. Consequently, hollow creation is slow, and in urban environments may be further limited by the removal of decaying tree limbs in the interests of public safety [20], [25], [26] and a reduction in fire frequency [27]–[29]. Thus the loss of critical resources (hollows) for breeding has the potential to strongly influence the abundance of cavity-nesting species in urban areas.

Despite the theoretical importance of hollow loss in urban areas [3], [30] there has been no study to date that has investigated the ecological impact on fauna. As a result, the link between urbanisation processes and changes in faunal community structure is not well understood. We used motion-triggered cameras during the breeding season to investigate hollow usage in urban remnants compared with that in undisturbed forest. We were particularly interested in differences in the assemblage of species using hollows, differences in visitation frequency and differences in the level of interference competition.

We predicted that.

1. visitation rates at hollows within suburban forest remnants will be higher than at hollows within undisturbed forest,
2. a greater diversity of parrots will visit individual hollows within suburban forest remnants than hollows within undisturbed forest and
3. there will be a greater level of interference interactions at hollows within suburban forest remnants than at hollows within undisturbed forest.

## Materials and Methods

### Ethics Statement

All animal work was conducted according to relevant national and international guidelines and was approved by the University of Sydney Animal Ethics Committee (approval number L04/9-2008/2/4896). This research was approved under New South Wales National Parks and Wildlife scientific license S12709. We also thank Royal National Park and Ku-ring-gai Chase National Park offices for their support as well as Bidjigal Reserve Trust, Sydney Olympic Park Authority, Parramatta City Council, Ku-ring-gai Council, Bankstown City Council, The Hills Shire Council and Sutherland Shire Council for allowing us to work on land within their jurisdiction.

### Study Sites

**Figure 1:**
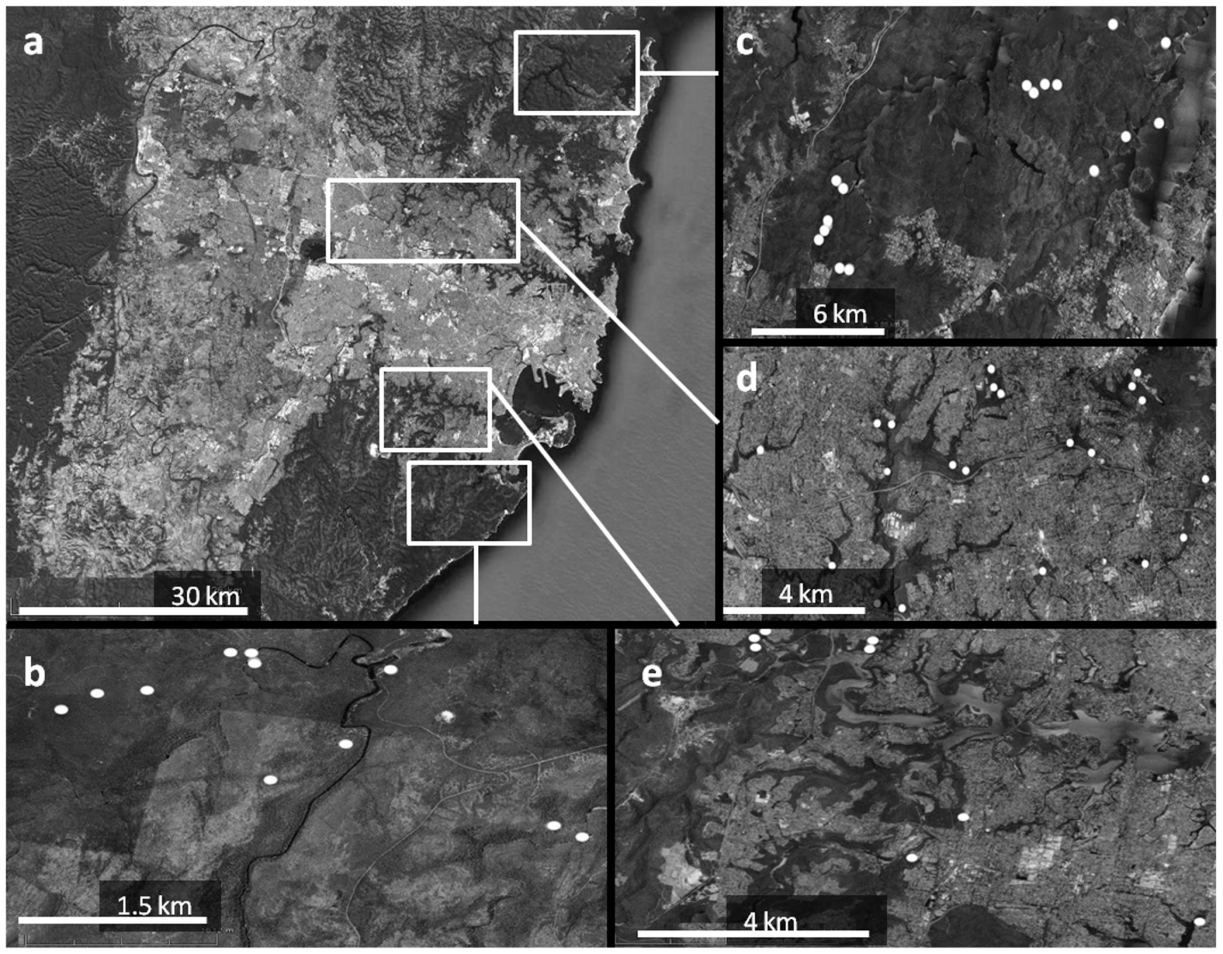
Map of Sydney, Australia showing a) Sydney region with the surrounding continuous forest National Parks to the north (Kur-ring-gai Chase National Park), south (Royal National Park) and west (Blue Mountains National Park). b) Camera locations within southern continuous forest. c) Camera locations in northern continuous forest. d) Camera locations in the northern half of Sydney. e) Camera locations in the southern half of Sydney. All images modified from Google Earth.

The study area encompassed the Sydney urbanised landscape on the east coast of New South Wales, Australia (Fig. 1a), bounded by the Pacific Ocean to the east and three major national parks to the north, south and west. It extends over an area greater than 12 000 square kilometres and is characterised by a warm, temperate climate.

Study sites in suburban remnant forest vegetation (hereafter referred to as ‘remnants’) (Fig. 1d, 1e) were selected from the suburban region of Sydney [6]. Candidate remnants were initially selected via satellite imagery and were required to meet the following criteria: 1) have an area greater than 2 ha (mean = 87.9 ha, se = ±29.74, min = 2.78, max = 475), 2) be at least 0.5 km apart from each other (mean = 1.32 km, se = ±0.26, min = 0.26, max = 4.83) and 3) be surrounded by housing. A sample of 22 remnants was randomly selected from the 44 that satisfied these criteria. Remnants differed in distance to undisturbed forest (mean = 20.77 km, se = ±2.60, min = 5.21, max = 39.68) and southern undisturbed forest (mean = 26.89 km, se = ±2.7, min = 6.22, max = 41.19). Sites in undisturbed forest (hereafter referred to as ‘forest’ (Fig. 1b, 1c) were selected from within Ku-ring-gai Chase National Park (13 500 ha) and Royal National Park (15 068 ha) to the north and south of Sydney respectively. They were chosen because they 1) were situated at similar distances from the coast as the remnant sites, 2) shared similar soil type and geology as the remnant sites, and 3) predominantly comprised Sydney Coastal or Sydney Hinterland Dry Sclerophyll or Sydney Forest [31].

### Tree Selection and Camera Installation

Hollow-bearing trees were selected from the genera *Angophora*, *Eucalyptus* and *Corymbia*, which comprise both the dominant canopy vegetation, and the main species of hollow-forming trees in the Sydney region. For a tree to be eligible for inclusion in the study, it had to meet five criteria. The tree must have contained a hollow visible from the ground that 1) was at least 3 m above the ground, 2) was in a part of the tree that was safe to access with climbing ropes, 3) was in a position where a camera could be fixed to view the hollow (see below for details), 4) had a minimum opening diameter of 3 cm and a minimum depth of 15 cm and 5) showed no signs of current occupation (presence of eggs, feathers or fur). These criteria for opening height, opening diameter and hollow depth were chosen based on minimum criteria that parrots have been observed to utilise [32], [33], and occupied hollows were avoided to ensure that we were not distracted by parental activity that might bias our otherwise random selection. The first hollow-bearing tree at each site, that met these criteria, was selected and hollows were accessed using “Single Rope Technique”, by which a slingshot was used to launch a temporary climbing rope into the tree that could then be ascended using climbing equipment. If, upon inspection, the hollow did not meet minimum width and depth criteria, the next tree that met these criteria was used. Thirty-two hollow-bearing trees from 22 remnants and 29 hollow-bearing trees in forest were selected. In some large remnants, several trees were selected, providing they were at least 0.5 km apart. Eighteen hollows were monitored for six months throughout June to November in 2009 and another sample of 43 hollows was monitored during the same months in 2010. This period included the breeding season of all local parrots (excluding the Yellow-tailed Black-Cockatoo) during 2009 and 2010 [32]. Data from the two years were pooled for analysis.

Once a tree-hollow had been selected, a single surveillance camera (Faunatech Scout Guard SG550V) was installed nearby. The camera was strapped to a branch or trunk that was either in front of, above, or to the side of the hollow, with the constraint that the camera could not impede access to the hollow. Cameras were positioned between 1 and 5 m from the entrance to the hollow and positioning of the camera depended upon tree and hollow morphology. Cameras were motion-triggered using passive infrared sensors with a 1 second shutter response time and a trigger range of up to 10 m. Twenty seconds of video footage were recorded each time the camera was triggered, during either day or night. An infrared LED flash was used at night so that no visible flash was produced. Two-gigabyte SD memory cards were used and cameras were inspected every three weeks to download data and replace batteries.

Each video was viewed and the species that triggered the camera, along with the time and date, were recorded. Footage arising from false triggers by wind-blown leaves was discarded. The cameras had a built-in delay of 2 seconds between subsequent video records, with the consequence that if an animal paused in front of a hollow for an extended period before entering, the moment of entry was not always recorded. Accordingly, all species that were recorded on camera in front of a hollow, in addition to species that were recorded actually entering or exiting the hollow, were included in subsequent analyses as hollow users. A control study, with cameras trained on hollows in hollow-bearing trees, as well as the branch/trunk of the nearest non-hollow-bearing tree of the same species and similar size, confirmed that negligible records of visits were made away from hollows (5.00±2.20 SE independent visits at hollow-bearing trees and 0.85±0.41 SE independent visits at non-hollow-bearing trees, *t*_(12)_ = 2.75, *p*<0.05).

### Hollow Measurements

Hollow type, opening diameter, opening height and depth of each hollow to be monitored were measured by climbing the tree. Types of hollows were classified as ‘trunk’, ‘pipe (a short section of hollowed residual branch that connects to the main trunk [34] or ‘branch’ [35] (Table 1). DBH, tree height and hollow height (height from the ground to the cavity entrance) were also recorded (Table 1). DBH was measured with a diameter tape and tree height and hollow height were measured with a Vertex Laser. Differences in hollow characteristics between remnants and continuous forest were determined by using independent t-tests. All variables met the assumption of normality as determined by Kolmogorov-Smirnov tests (*p*>0.05).

**Table 1:** Characteristics of monitored hollows and trees that contained the hollow.

Hollow Characteristic	Remnant(n = 32)	Forest(n = 29)
Hollow Entrance Length (cm)	19.75±2.24	19.07±2.45
Hollow Entrance Width (cm)	15.58±1.76	15.57±1.85
Hollow Depth (cm)	87.09±12.67	56.77±8.69
Tree Height (m)	21.57±1.47	17.04±1.39
Hollow Height from ground (m)	10.80±0.75	9.37±0.81
Hollow Type (number present)		
Pipe	11	14
Trunk	17	10
Branch	4	5

### Community Structure of the Hollow-using Assemblage

Species utilising each tree hollow were identified and differences in the structure of the hollow-using assemblage between remnants and forest were analysed with multidimensional scaling using the PRIMER (version 5.2) statistical package [36]. For community structure analyses, presence/absence data were used in order to eliminate the influence of multiple visits by the same individual. If a species was recorded once throughout the entire recording period for a particular hollow, it was recorded as present. The percentage contribution of each species to the differences in community structure of visitors, between hollows in remnants and forest, were then determined using the SIMPER routine in PRIMER and species evenness was displayed with a rank abundance graph. The multivariate dispersion (variation among assemblages within each habitat, measured by the deviations from centroid) between remnants and forest was compared using the PERMDISP function in PRIMER (version 6.1.6).

### Species Visitation, Diversity and Nest Occupancy

The automatically-triggered cameras frequently recorded several segments of footage from the same visit or multiple visits on a single day from the same species. To remove this source of bias we analysed “independent visits”, which we defined as a single visit per species per day that did not include the ‘owner’ of the hollow. Ownership of a hollow was assigned to a species if that species was responsible for in excess of 50% of the total number of visitations made by all species to the hollow. The total number of independent visits by each species was then divided by the total number of days that the camera was recording, to generate an index of visitation, which corrected for differences in recording time between cameras. Most species were recorded infrequently, and so for the purpose of statistical analysis the records of some species were pooled into groups. Thus the crimson rosella, Australian king parrot, eastern rosella, musk lorikeet, scaly-breasted lorikeet and yellow-tailed black-cockatoo were combined into a single ‘Other Parrot’ variable. (Scientific names of all species throughout the manuscript are given in Table 2.) The common brushtail possum, lace monitor, common ringtail possum, eastern pygmy possum, sugar glider, squirrel glider, feathertail glider, white-throated treecreeper, laughing kookaburra, grey shrike-thrush, Australian wood duck, southern boobook and powerful owl were also pooled into a single ‘Other Fauna’ variable for statistical analysis. The rainbow lorikeet and sulphur-crested cockatoo were recorded frequently enough to be analysed as separate variables. The rainbow lorikeet, sulphur-crested cockatoo, ‘Other Parrots’ and ‘Other Fauna’ variables were transformed with either a log or square root transformation. Assumptions of normality were checked by assessing skewness and kurtosis values [37].

**Table 2:** The number of independent visitations of taxa recorded at hollows in both remnants and continuous forest and whether or not they are known to use hollows.

Species	Class	Hollow Usage	Independent Visitations Remnants	Independent Visitations Forest	Average Body Length (cm)
Pied butcherbird (*Cracticus nigrogularis)*	Aves	No	13	5	35
Sulphur-crested cockatoo (*Cacatua galerita)*	Aves	Yes (Barnard, 1914)	145	200	48
Rainbow lorikeet (*Trichoglossus haematodus)*	Aves	Yes (Lamont, 1997)	522	49	30
Crimson rosella (*Platycercus elegans*)	Aves	Yes (Hyem, 1936)	31	7	34
Australian raven (*Corvus coronoides*)	Aves	No	1	0	52
White-throated treecreeper (*Cormobates leucophaeus*)	Aves	Yes (Higgins et al., 2001)	2	2	15
White-browed wood swallow (*Artamus superciliosus*)	Aves	Yes (LaSouef, 1903)	1	0	20
Australian king parrot (*Alisterus scapularis*)	Aves	Yes (Favaloro, 1931)	14	3	42
Galah (*Cacatua roseicapilla*)	Aves	Yes (Higgins, 1999)	40	0	36
Laughing kookaburra (*Dacelo novaeguineae*)	Aves	Yes (Hindwood, 1959)	25	0	42
Noisy miner (*Manorina melanocephala*)	Aves	No	32	0	26
Eastern rosella (*Platycercus eximius*)	Aves	Yes (Higgins, 1999)	17	0	30
Powerful owl (*Ninox strenua*)	Aves	Yes (Gibbons, 1989)	4	2	55
Southern boobook (*Ninox novaeseelandiae*)	Aves	Yes (Bryant, 1941)	0	1	29
Grey shrike-thrush (*Colluricincla harmonica*)	Aves	Yes (Higgins and Peter, 2002)	0	7	24
Musk lorikeet (*Glossopsitta concinna*)	Aves	Yes (Higgins, 1999)	4	0	22
Australian wood duck (*Chenonetta jubata*)	Aves	Yes (Frith, 1982)	16	9	47
Yellow-tufted Honeyeater (*Lichenostomus melanops*)	Aves	No	0	2	11
Scaly-breasted lorikeet (*Trichoglossus chlorolepidotus*)	Aves	Yes (Higgins, 1999)	1	0	23
Yellow-tailed black-cockatoo (*Calyptorhynchus* *funereus*)	Aves	Yes (Higgins, 1999)	0	1	60
European honey bee (*Apis mellifera*)	Insecta	Yes (Oldroyd et al., 1994)	N/A	N/A	1.6
Eastern pygmy possum (*Cercartetus nanus*)	Mammalia	Yes (Jones and Parish, 2006)	0	29	90
Common brushtail possum (*Trichosurus vulpecula)*	Mammalia	Yes (Jones and Parish, 2006)	195	11	450
Common ringtail possum (*Pseudocheirus peregrinus*)	Mammalia	Yes (Jones and Parish, 2006)	11	13	325
Sugar glider (*Petaurus breviceps*)	Mammalia	Yes (Jones and Parish, 2006)	5	12	185
Squirrel glider (*Petaurus norfolcensis*)	Mammalia	Yes (Jones and Parish, 2006)	0	6	205
Feathertail glider (*Acrobates pygmaeus*)	Mammalia	Yes (Jones and Parish, 2006)	0	27	73
Lace monitor (*Varanus varius)*	Reptilia	Yes (Russell et al., 2003)	0	21	55
Skink (*Scincidae*)	Reptilia	Yes (Munks et al., 2007)	8	8	Varies with species

Differences in visitation rate between hollows in remnants and forests, and between the four taxon variables, were analysed using a two-factor ANOVA. As significant interactions existed, post-hoc Scheffé controlled contrasts were then used to further explore differences between habitat and fauna.

Species diversity at each hollow was calculated with the Shannon Wiener Diversity Index [38], [39] using the number of independent visits. The difference in mean species diversity between remnant and forest hollows was then tested using a t-test.

During climbed inspections, hollows within both remnants and forest that had either eggs or chicks present were deemed to be occupied nests and the identity of the parents was inferred both from the species of chick and video footage at the nest entrance. It was not possible to determine if all nests contained eggs or chicks, as some hollows were too deep to observe the cavity floor.

### Species Interactions at Hollows

At some hollows interactions both between species, and within species, were recorded in the video footage. Both interspecific and intraspecific interactions were divided by the number of recording days for each hollow and were compared between remnants and forests using a t-test. These data did not meet the assumption of normality as determined by the Kolmogorov-Smirnov test (*p*>0.05), however skewness values (1.54) and kurtosis values (1.14) were deemed acceptable and the t-test was run with alpha adjusted to 0.01 [37].The number of intraspecific interactions compared to interspecific interactions was compared for both the rainbow lorikeet and the sulphur-crested cockatoo using a Chi-square test. Interspecific interactions were compared for both the sulphur-crested cockatoo and the rainbow lorikeet. Species interactions were classified as either an attack or a defence. Successful attacks occurred when an individual executing the attack successfully displaced the individual who was present at the hollow at the time of attack. Attack behaviours usually consisted of swooping, lunging, charging or fighting. Attacks were considered unsuccessful if the attacker failed to displace the individual present at the hollow at the time of attack. A successful defence occurred when the individual at the hollow was able to remain at the hollow when under attack, whereas an unsuccessful defence resulted in the individual being displaced from the hollow. The number of attacks and defences was compared using Chi-Square tests for both the rainbow lorikeet and the sulphur-crested cockatoo respectively. Chi-Square tests were then used to compare the successful and unsuccessful execution of the most frequent behaviour (either attack or defence) for the sulphur-crested cockatoo and the rainbow lorikeet respectively.

## Results

A total of 11 879 episodes of visitation was recorded from the 61 hollows during 5401 camera-days of recording. Thirty-one species were detected, of which 23 are known to use hollows for nesting (Table 2). Occupying and visiting species comprised mammals (6 species), birds (14 species) reptiles (2 species) and insects (one species), and of the birds, 9 species were parrots. Whilst insects were not included in analyses, it should be noted that European honey bees were present at 6 out of 61 hollows. Using the definition of an independent visit as a daily visit of a particular species that was not an ‘owner’ of a hollow, 1502 independent visits were recorded.

### Hollow Measurements

Hollow depth in remnants was significantly greater compared to hollows in continuous forest (t_59_ = 2.11, p<0.05) (Table 1). Hollow-bearing trees within remnants were also significantly taller than hollow-bearing trees within continuous forest (t_59_ = 2.53, p<0.05) (Table 1). There was no significant difference in either the height of cavities from the ground or hollow entrance dimensions in remnants compared to forest (Table 1).

### Community Structure and Species Diversity

**Figure 2:**
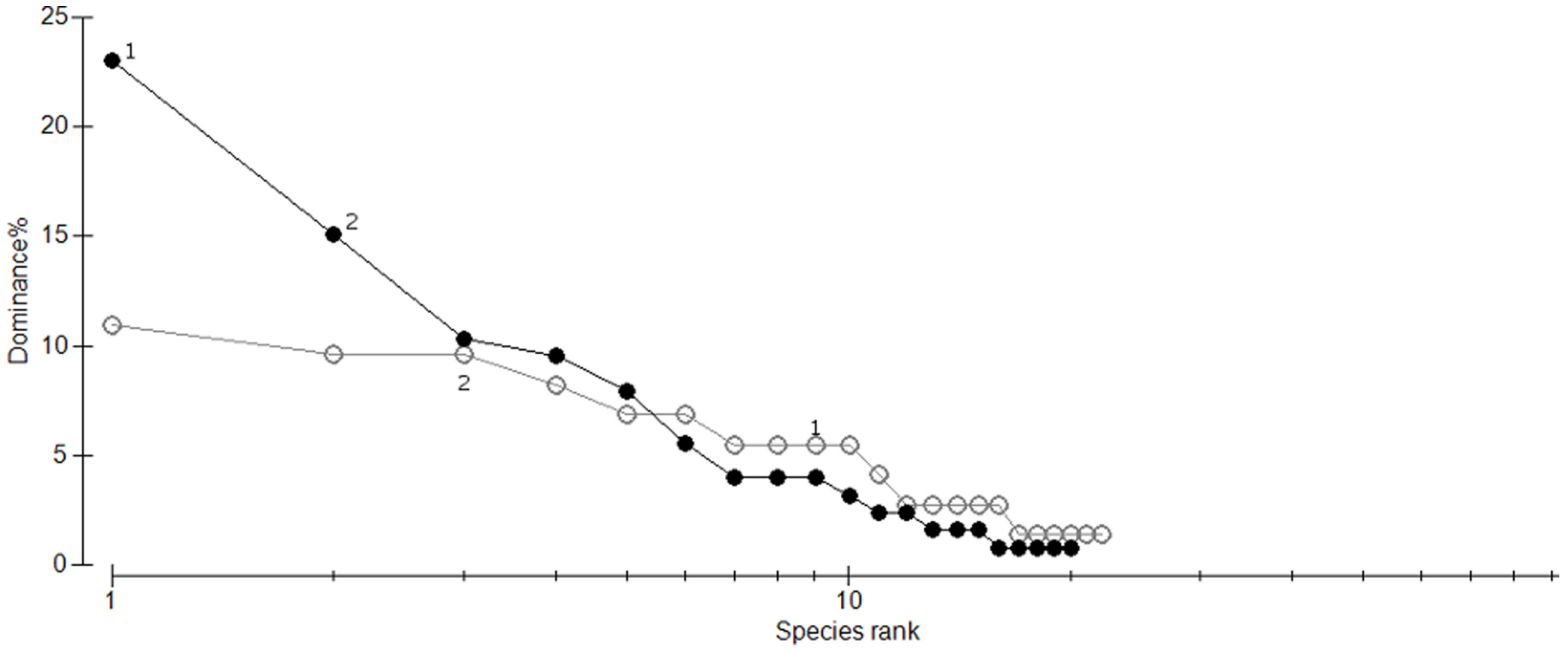
Rank abundance curve of independent visits to hollows in remnants (black line with closed circles) and continuous forest (grey line with open circles). ‘1’ denotes the rainbow lorikeet. ‘2’ denotes the sulphur-crested cockatoo.

**Figure 3:**
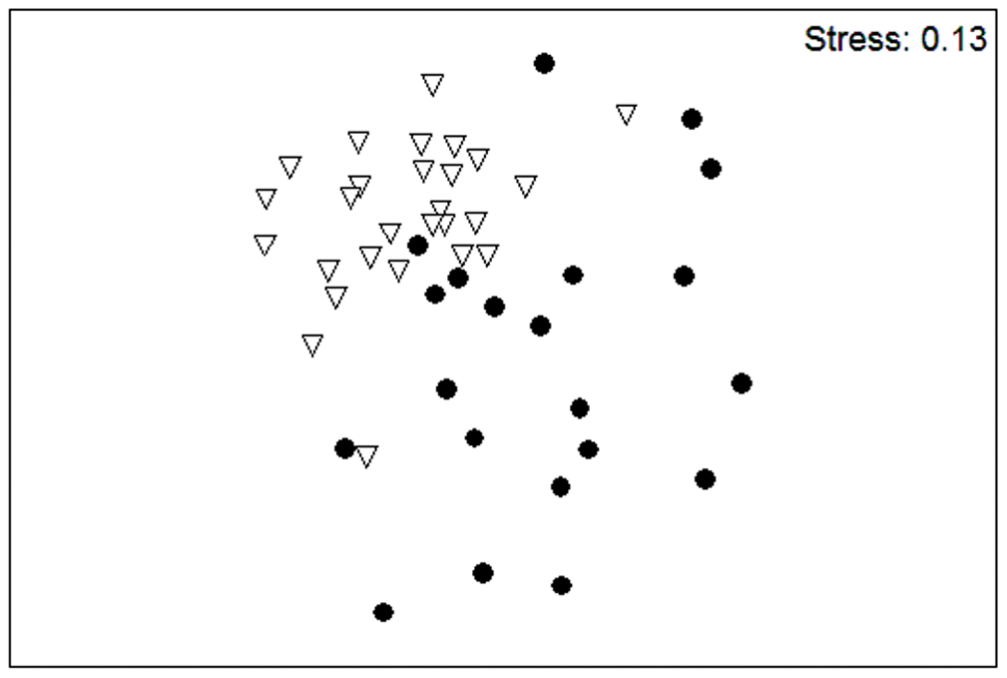
Multi-dimensional scaling plot showing the differences in the fauna assemblage between hollows in remnants (open triangle) hollows in continuous forest (filled circle).

Hollows within remnants had a significantly different assemblage of occupying and visiting species compared to hollows in forest (*Global R* = 0.457, *p*<0.05). Both the rainbow lorikeet and the sulphur-crested cockatoo were the most characteristic species associated with hollows in remnants, accounting for 56% and 19% of the within-habitat similarity, but they contributed only 3% and 12% of the similarity in the hollow-using assemblage in forest (Fig. 2). The high abundance of the rainbow lorikeet at hollows in remnants is primarily responsible for the steep decline in relative abundance, indicating an uneven species diversity of the hollow-utilising community within remnants (Fig. 2). The pygmy possum, lace monitor and sulphur-crested cockatoo characterised visitors to hollows in forest, collectively contributing 54% of the within-habitat similarity and greater species evenness. The composition of the assemblage of hollow users within forest was significantly more variable than the assemblage-using hollows within remnants (*F*_1, 50_ = 22.66, p<0.01) as is indicated by the wider spread of points in Fig. 3.

### Visitation

**Figure 4:**
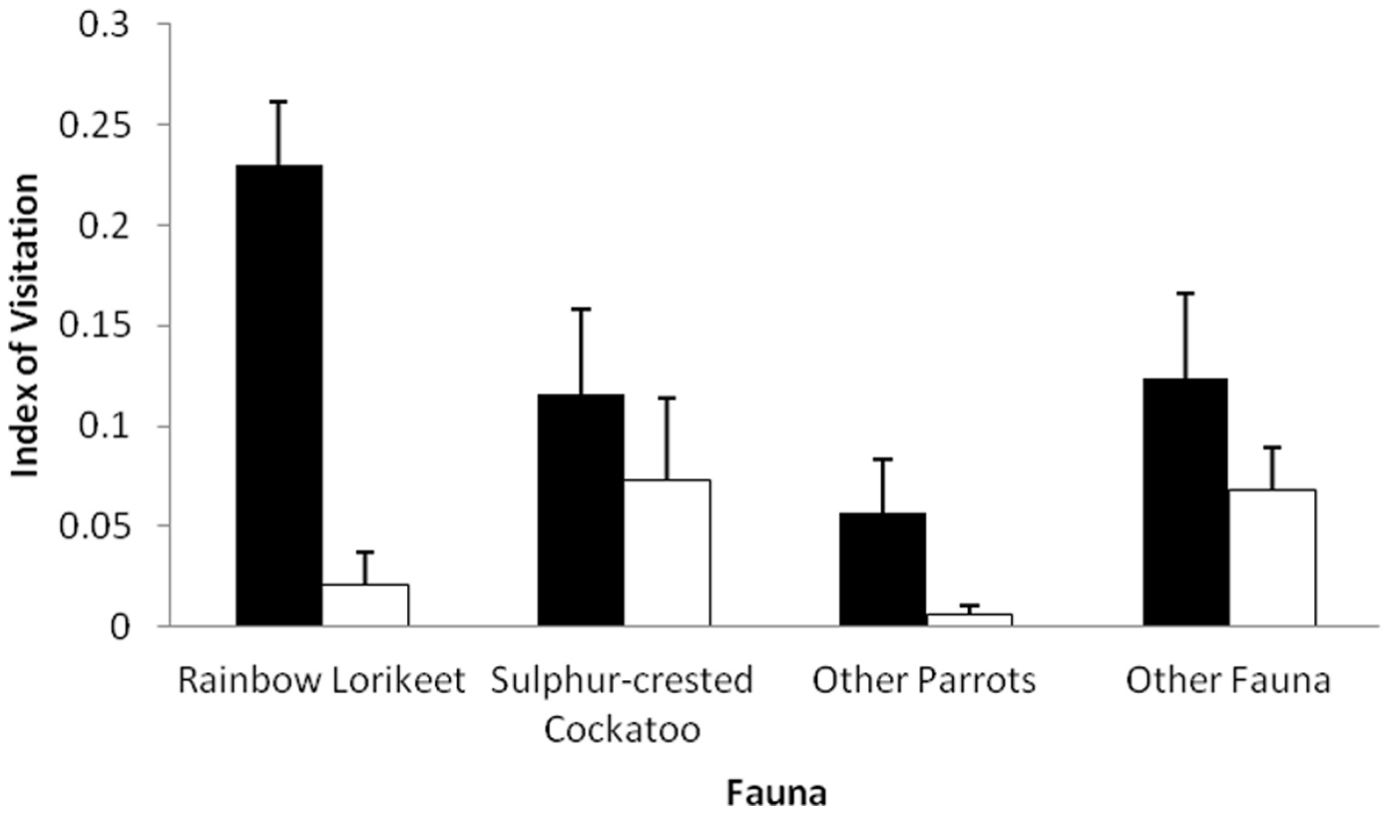
Index of visitation for the rainbow lorikeet, sulphur-crested cockatoo, Other Parrots and Other Fauna at hollows in urban remnants (closed bars) and hollows in continuous forest (open bars). Index of visitation was calculated by dividing the number of independent visits (one visit per species per day) by the number of days the camera was recording.

Hollows in remnants had significantly more faunal visitations than did hollows in forest, with a visitation rate of 0.074±0.035 (se) independent visits (exclusive of owners) per day at hollows in remnants compared with a visitation rate of 0.027±0.017 (se) at hollows in forest (*F*_1, 59_ = 21.35, *p*<0.05). As expected, the visitation rate differed significantly between the arbitrary taxon groupings (*F_3, 59_* = 4.03, *p*<0.01), however the more important interaction between habitat and fauna was also significant (*F_3, 59_* = 4.46, *p*<0.01). Using Scheffè controlled comparisons, hollows within remnants had significantly more visitations from both rainbow lorikeets (F_1, 59_ = 78.05, p<0.01) and Other Parrots (F_1, 59_ = 11.13, p<0.01) than did hollows in forest (Fig. 4). There was no difference in visitation rate between hollows in remnants and hollows in forest for either the sulphur-crested cockatoo or Other Fauna. Furthermore, hollows within remnants had significantly more visitations by rainbow lorikeets than by sulphur-crested cockatoos, Other Parrots and Other Fauna combined (*F*_3, 59_ = 88.54, *p*<0.01) (Fig. 4). Other Fauna made significantly more visitations to hollows in both remnant and forest than did Other Parrots (*F*_1, 59_ = 13.86, *p*<0.01) (Fig. 4).

There was no significant difference in species diversity, as expressed in terms of the Shannon Wiener diversity index (t_(59)_ = 1.32, p>0.05), between hollows within remnants (2.39±0.17 se) and forest (2.01±0.24 se).

In hollows within remnant vegetation where the hollow floor was visible, eggs were observed in two hollows and sulphur-crested cockatoo chicks were observed in one hollow. One of the clutches of eggs belonged to a pair of galahs. The identity of the parents of the second clutch was unable to be determined and the eggs appeared to have been abandoned. Neither the galah eggs nor sulphur-crested cockatoo chicks were present at the next inspection three weeks later. In hollows within forest where the hollow floor was visible, eggs were present within one hollow, but they were not present at the next inspection. Lace monitors were observed visiting the hollow and most likely preyed on the eggs. One other hollow within continuous forest was observed with a nestling, and a juvenile cockatoo eventually fledged from this hollow.

### Species Interactions

One hundred and thirty-seven aggressive interactions were recorded across all hollows in both remnants and forest, involving eight species of birds and one species of mammal, and comprising both interspecific and intraspecific interactions. There were significantly more interactions per hollow per day at remnant hollows (mean = 0.081±0.025 (se)) than at hollows in forest (mean = 0.053, ±0.021 (se)) (*t*_(59)_ = 2.39, p<0.05).

**Figure 5:**
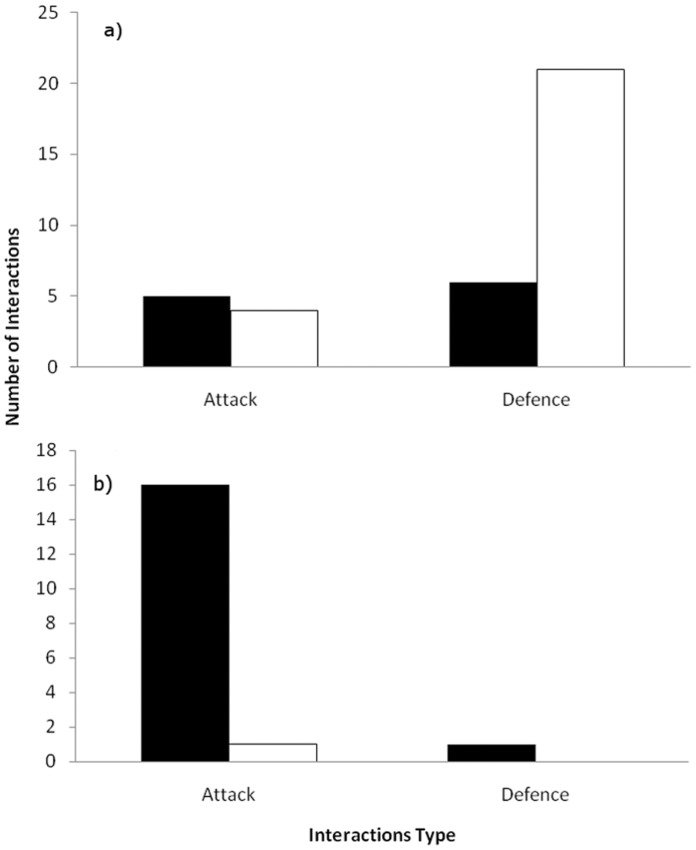
Number of aggressive interactions observed at hollows in both urban remnants and continuous forest for the a) rainbow lorikeet and the b) sulphur-crested cockatoo separated into attacks and defences. Closed bars denote successful interactions. Open bars denote unsuccessful interactions.

Significantly more intraspecific interactions (n = 70) than interspecific interactions (n = 37) were recorded for the rainbow lorikeet (*χ* ^2^_(1)_ = 10.18, *p*<0.01). Interspecific interactions (Table 3) involving the rainbow lorikeet were comprised of significantly more defences than attacks (*χ* ^2^_(1)_ = 9.0, *p*<0.01), of which significantly more defences were unsuccessful (*χ* ^2^_(1)_ = 8.33, *p*<0.01) (Fig. 5), with the rainbow lorikeet failing to defend against the sulphur-crested cockatoo, the galah and the Australian wood duck. The rainbow lorikeet successfully defended against the laughing kookaburra and the noisy miner, as well as one successful defence against the sulphur-crested cockatoo. The rainbow lorikeet successfully attacked the pied butcherbird, Australian king parrot, eastern rosella, and the galah, but was unsuccessful when attempting to attack the sulphur-crested cockatoo, as well as once against the galah and the eastern rosella.

**Table 3:** The number of aggressive interactions recorded within and between species pooled for urban remnants and continuous forest.

*Species*	*Sulphur-crested Cockatoo*	*Rainbow Lorikeet*	*Eastern Rosella*
*Sulphur-crested cockatoo*	27	14	0
*Rainbow lorikeet*	17	70	3
*Galah*	0	9	0
*Australian king parrot*	0	1	1
*Laughing kookaburra*	0	1	0
*Crimson rosella*	1	0	0
*Pied butcherbird*	0	1	0
*Noisy miner*	0	3	0
*Common brushtail possum*	1	0	0
*Australian wood duck*	0	1	0
*Unidentified*	1	0	0

The sulphur-crested cockatoo made significantly more attacks than defences (*χ* ^2^_(1)_ = 14.22, *p*<0.01), of which significantly more attacks were successful than unsuccessful (*χ* ^2^_(1)_ = 13.24, *p*<0.01) (Fig. 5). Successful attacks were made against the rainbow lorikeet, crimson rosella and the common brushtail possum. One unsuccessful attack was made against the rainbow lorikeet. There was no significant difference in the number of intraspecific attacks.

## Discussion

Tree hollows within urban remnants had a significantly different assemblage of visiting taxa than hollows within continuous forest, with parrots, in particular the rainbow lorikeet, making significantly more visitations than other taxa to hollows within remnants. The high rate of visitation to urban hollows compared to hollows within forest may be associated with high densities of rainbow lorikeets and other parrots within the urban region [6], [21], [40]. Alternatively, it may be indicative of a shortage of suitable nesting cavities within urban remnants.

The number of interactions at hollows within remnants was significantly higher than at hollows within forest and the number of intraspecific interactions between rainbow lorikeets was significantly higher than interspecific interactions between rainbow lorikeets and other taxa. High rates of intraspecific interactions at hollows have previously been observed in habitat with limited cavity availability [10]–[12], [14] and significantly fewer hollow-bearing trees have been observed within Sydney remnant vegetation (2.8 ha^−1^) than in continuous forest (6.5 ha^−1^) (Davis, unpubl. data). Additionally within the urban region, hollow-bearing trees tend to be ‘clumped’ within remnants, which has been previously associated with a higher number of intraspecific interactions, as species attempt to maintain nesting trees or territories [41].

A high number of intraspecific interactions has also been associated with competition for high quality nesting sites [42] and may not necessarily indicate a lack of available hollows. When a diverse supply of suitable nesting hollows is present, species may choose hollows with characteristics specific to their body size or breeding requirements [35], [43], [44]. Remnants contained significantly more hollows that were deeper and present in the main trunk of the tree, and in trees that were taller than those in continuous forest. As they are large birds, cockatoos need hollows large enough to provide shelter and to rear nestlings, and have often been recorded using hollows that occur within the main trunk of the tree [32], [33], [45]. The higher number of main trunk cavities in remnants may therefore be sufficient to support the population of sulphur-crested cockatoos within the urban region. The lack of a significant difference between the number of intraspecific and interspecific interactions for the cockatoo may further support this. The high number of intraspecific interactions for the rainbow lorikeet may suggest that optimal hollows for this species are in lower abundance than those suited to sulphur-crested cockatoos.

As previously noted, the higher abundance of the rainbow lorikeet and other parrot species in the urban region compared to continuous forest [6], coupled with the high number of intraspecific interactions between rainbow lorikeets, the high number of interactions in remnants may be due to an inadequate supply of suitable hollows.

A shortage of suitable nesting hollows is capable of influencing faunal assemblage composition through competition. Interference competition may limit availability of and access to nesting hollows for breeding pairs, particularly in habitat with clumped nesting hollows, as nesting pairs of some species defend territory around the nest or attempt to maintain multiple cavities suitable for nesting ([13], [16]). High levels of interference competition may explain the low number of nesting attempts in this study, as nest establishment or breeding attempts are known to fail due to a greater investment in nest defence [12], [19], [46], [47]. Alternatively, low numbers of nesting attempts may either be due to hollows selected for the study having sub-optimal characteristics or due to some parrots maintaining several potential nesting hollows within their territory [13], [16].

Whilst there is currently no evidence of a decline in urban parrot diversity, the urban avian community may still be reaching equilibrium [48]. Rainbow lorikeets have only recently established in southern Sydney and, should they continue to further increase in both density and abundance, there is the possibility that this may lead to the exclusion of less competitive parrots [49].

There is a need for wildlife managers to understand the complex relationship between human activities, subsequent habitat modification and biodiversity decline. Potential shortages in either hollow availability or suitable nesting sites and a subsequently higher number of competitive interactions at hollows may represent an increasing threat to biodiversity. In addition, increasing densities of certain native species may pose a threat that is greater than that of exotic species such as the common myna, which is commonly perceived to have a detrimental impact on native cavity-nesting wildlife [50], [51], and consequently being the focus of costly eradication programs. Interestingly, common mynas, were not recorded at any tree hollows in this study, and the species is not likely to be a structuring force in this urban assemblage. While a common species in Sydney, and the subject of much anecdotal discussion in terms of it’s possible impact, its effect may well be completely confined to urban parks and suburban gardens [51], [52].

Further research is needed into urban hollow usage, particularly to determine the availability of hollows as well as if, and the extent to which, hollows may be limiting within urban environments. More data are required to determine species-specific preferences for hollows with particular characteristics as well as information on the reproductive success of parrots in urban remnants. Finally, the conservation of urban wildlife is integral to ensuring that people living within cities maintain both an appreciation of wildlife and recognise the value of wildlife conservation.
